# Characterization of the Phosphotransferase from *Bacillus subtilis* 1101 That Is Responsible for the Biotransformation of Zearalenone

**DOI:** 10.3390/toxins17010021

**Published:** 2025-01-03

**Authors:** Yuzhuo Wu, Qiuyu Zhou, Junqiang Hu, Yunfan Shan, Jinyue Liu, Gang Wang, Yin-Won Lee, Jianrong Shi, Jianhong Xu

**Affiliations:** 1 School of Food and Biological Engineering, Jiangsu University, Zhenjiang 212013, China; 2222218095@stmail.ujs.edu.cn (Y.W.); wanggang2015@jaas.ac.cn (G.W.); 2 Jiangsu Key Laboratory for Food Quality and Safety-State Key Laboratory Cultivation Base, Ministry of Science and Technology, Key Laboratory for Agro-Product Safety Risk Evaluation (Nanjing), Ministry of Agriculture and Rural Affairs, Key Laboratory for Control Technology and Standard for Agro-Product Safety and Quality, Ministry of Agriculture and Rural Affairs, Collaborative Innovation Center for Modern Grain Circulation and Safety, Institute of Food Safety and Nutrition, Jiangsu Academy of Agricultural Sciences, Nanjing 210014, China; mz120221326@stu.yzu.edu.cn (Q.Z.); 2021216027@stu.njau.edu.cn (J.H.); 2023816130@stu.njau.edu.cn (Y.S.); 232712053@njnu.edu.cn (J.L.); lee2443@snu.ac.kr (Y.-W.L.); shiji@jaas.ac.cn (J.S.); 3 College of Agriculture, Yangzhou University, Yangzhou 225009, China; 4 College of Life Science, Nanjing Agriculture University, Nanjing 210095, China; 5 School of Food Science and Pharmaceutical Engineering, Nanjing Normal University, Nanjing 210023, China; 6 School of Agricultural Biotechnology, Seoul National University, Seoul 08826, Republic of Korea

**Keywords:** zearalenone, biotransformation, *Bacillus subtilis*, phosphotransferase

## Abstract

*Bacillus* microorganisms play an important role in the zearalenone (ZEA) biotransformation process in natural environments. The phosphotransferase pathway in *Bacillus* is both widespread and relatively well conserved. However, the reaction kinetics of these phosphotransferases remain poorly understood, and their catalytic activities are suboptimal. In this study, a ZEA phosphotransferase, ZPH1101, was identified from *Bacillus subtilis* 1101 using genome sequencing. The product transformed by ZPH1101 was identified as phosphorylated ZEA (ZEA-P) through LC-TOF-MS/MS analysis. The experiments conducted on MCF-7 cells demonstrated that ZEA-P exhibited a lower level of estrogenic toxicity than ZEA. The optimal reaction conditions for ZPH1101 were determined to be 45 °C and pH 8.0. The maximum velocity (*V*_max_), Michaelis constant (*K*_m_), and catalytic constant (*k*_cat_) were calculated through fitting to be 16.40 μM·s^−1^·mg^−1^, 18.18 μM, and 54.69 s^−1^, respectively. Furthermore, adding 1 mmol/L Fe^2+^ or Fe^3+^ to the reaction system increased the efficiency of ZPH1101 in converting ZEA by 100% relative to the system containing solely 1 mmol/L ATP and 1 mmol/L Mg^2+^, suggesting that low concentrations of Fe^2+^ or Fe^3+^ can improve the ZPH1101-mediated transformation of ZEA. This study contributes to the enzymatic removal of ZEA and broadens the spectrum of strain and enzyme options available to researchers for ZEA detoxification efforts.

## 1. Introduction

Zearalenone (ZEA) is a secondary metabolite that is produced by a variety of *Fusarium* species. It is recognized for its estrogenic effects and detrimental impact on health. ZEA is a common contaminant of grains, including maize, wheat, barley, and rice, as well as their processed products. It demonstrates substantial toxicity to the reproductive system, liver, and kidneys in both animals and humans [1,2,3]. Currently, the principal strategies for ZEA elimination include physical, chemical, and biological approaches [4]. However, most physical and chemical methods have limitations, such as the loss of nutrients, low efficacy in detoxification, and limited selectivity [4,5,6]. Consequently, biological enzyme detoxification has gained significant interest in recent years due to its high selectivity, mild operating conditions, and low effect on the sensory properties of raw materials [7,8]. The key to this procedure is the use of enzymes produced by microorganisms to eliminate ZEA. This approach not only transforms toxins into non-toxic or less toxic compounds by altering their structure but also offers an environmentally friendly and sustainable solution [9]. It is now widely recognized as the most promising technology for ZEA detoxification [10].

To date, researchers have identified many fungi and bacteria that are capable of degrading ZEA. Takahashi-Ando et al. isolated *Clonostachys rosea* IFO 7063 and cloned its esterase gene *zhd101* [11]. Their study demonstrated that ZHD101 has the ability to cleave lactone bonds, resulting in the breakdown of ZEA. Additionally, various bacterial species capable of degrading ZEA have been identified, including *Aeromicrobium* [12], *Rhodococcus* [13], *Acinetobacter* [14,15], *Pseudomonas* [16,17], *Lactobacillus* [18], and *Bacillus* [19,20,21,22,23,24,25]. Among these, *Bacillus* is the most frequently reported bacterium for ZEA degradation or modification, with *B. velezensis* and *B. amyloliquefaciens* being the most prevalent. However, only a limited number of specific probiotic *Bacillus* species are appropriate for use in the feed industry, owing to severe safety and regulatory criteria, including *B. subtilis* [21,22], *B. licheniformis* [23,24], and *B. pumilus* [25]. These strains must be proven harmless to both animals and humans, produce no toxic or harmful metabolites, and be certified by relevant international or national authorities. Thus, isolating and screening of *Bacillus* strains capable of efficiently degrading ZEA and suitable for direct application as microbial additives are critical to the feed industry.

Various enzymes in *Bacillus* have been demonstrated to catalyze the biotransformation of ZEA, including glycoside transferases [26,27], laccases [24,28], and phosphotransferases [29]. There has been little research on ZEA phosphotransferase, with publications exclusively by Zhu et al. and Yang et al. [29,30]. None of the studies investigated the biochemical characteristics, catalytic sites, or mechanisms of ZEA phosphotransferase, emphasizing the necessity for additional exploration and examination of this enzyme. This study aimed to isolate probiotics capable of efficiently degrading ZEA for application in the feed industry, to elucidate the degradation or biotransformation mechanism of ZEA from an enzymatic perspective, and to establish a foundation for the subsequent industrial application of the enzyme. This study contributes our understanding of the phosphotransferase pathways involved in ZEA biotransformation by *B. subtilis*.

## 2. Results

### 2.1. Isolation, Identification, and Characteristics of Strain 1101

**Figure 1:**
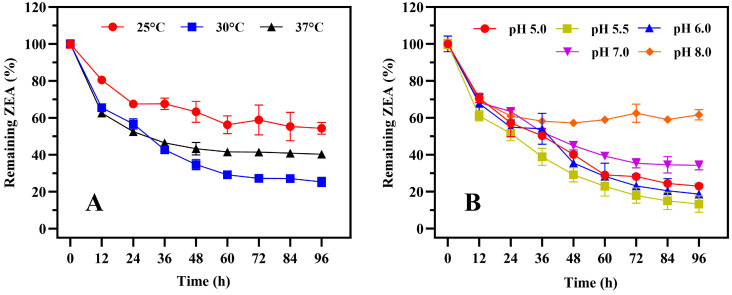
The biotransformation characteristics of strain 1101 under various conditions. ZEA biotransformation curves of strain 1101 at different temperatures (**A**) and pH levels (**B**), where the minimal salt medium containing 10 μg/mL ZEA was used to determine the biotransformation efficiency of strain 1101. Error bars represent standard deviations.

A total of 115 bacterial strains were isolated from livestock feces, of which three showed ZEA elimination capacity, with one strain displaying significantly higher elimination activity than the other two strains, and was thus selected for further research. The strain’s colonies on LB plates displayed a rough and opaque surface, with colors ranging from milky white to slightly yellow (Figure S1A). The specific *Bacillus* species was identified using a combination of 16S rRNA and *gyrB* gene sequences [31], with the primer pairs listed in Table S1. The detected bands for the 16S rRNA and *gyrB* genes were consistent with their expected sizes (Figure S1B). Based on morphological observation and phylogenetic analysis (Figure S1C), the strain was identified as *Bacillus subtilis*, designated as 1101. We determined that the biosorption of ZEA by *B. subtilis* 1101 was negligible based on the elimination ability of strain 1101 to ZEA tested under different treatment conditions (Figure S1D). The results indicate that the primary role in ZEA elimination is attributed to the intracellular enzyme activity. The optimal growth temperature and pH ranges for *B. subtilis* 1101 were determined to be 30–37 °C and 5.0–7.0, respectively (Figure S2A,B). The optimal temperature for biotransformation was shown to be 30 °C, with an optimal pH of 5.5 (Figure 1A,B).

### 2.2. Expression and Purification of Recombinant ZPH1101

The *zph1101* gene was discovered in the genome of *B. subtilis* 1101 by comparing its homology and annotating the gene using ZPH (accession number: QVL29807) as the template. We found 100% homology of ZPH1101 to a putative phosphotransferase from *B. subtilis* QB928 (accession number: AFQ59371). The sequence alignment revealed a 69% amino sequence similarity between ZPH from *B. subtilis* Y816 and ZPH1101. The recombinant enzyme ZPH1101 was expressed in *E. coli* BL21(DE3) as a His-tagged fusion protein. Following induction with IPTG, the cells were harvested and lysed to extract the target protein. The His-tagged ZPH1101 was purified using Ni-NTA affinity chromatography, ensuring high purity. Subsequent dialysis was performed to remove imidazole and prepare the protein for downstream applications. These steps collectively ensured the production of a functional recombinant enzyme. The purified recombinant ZPH1101 was determined by SDS-PAGE, showing a single band at 90 kDa (Figure S3), which corresponds to the theoretical molecular weight of the recombinant protein.

### 2.3. Biotransformation of ZEA by Recombinant ZPH1101

**Figure 2:**
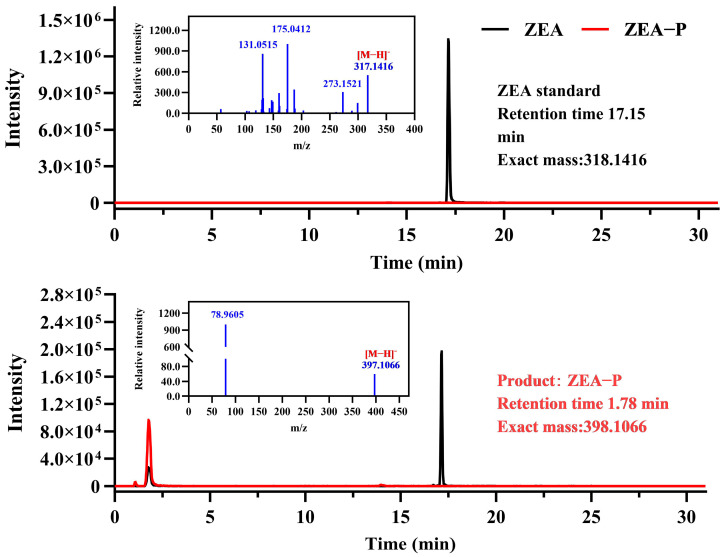
LC-TOF-MS/MS analysis of ZEA biotransformation products (ZEA-P) following incubation with purified recombinant ZPH1101. Secondary mass spectrometry data indicated that the negative ion ESI-MS spectrum displayed significant fragment ions, including deprotonated ZEA at *m*/*z* 317.1416 ([M-H]^−^), the phosphate group at *m*/*z* 78.9605 ([M-H]^−^), and the phosphorylated product at *m*/*z* 397.1066 ([M-H]^−^).

To clarify the catalytic mechanism of ZPH1101 on ZEA, the products were analyzed following incubation of ZEA and purified recombinant ZPH1101. Employing a highly sensitive LC-TOF-MS/MS system, the analysis of negative ion ESI-MS data revealed critical fragment ions of deprotonated ZEA (*m*/*z* 317.1416 [M-H]^−^), phosphate group (*m*/*z* 78.9605 [M-H]^−^), and product (*m*/*z* 397.1066 [M-H]^−^) (Figure 2). The chemical formula of the biotransformation product was determined to be C_18_H_24_O_8_P, which indicates that the product is phosphorylated ZEA (ZEA-P). Based on LC-TOF-MS/MS fragmentation pattern, we deduced that the phosphorylation catalyzed by ZPH1101 involves the substitution of a hydroxyl group at either the C14 or C16 position of ZEA with a single phosphate group.

### 2.4. Characterization of Recombinant ZPH1101

**Figure 3:**
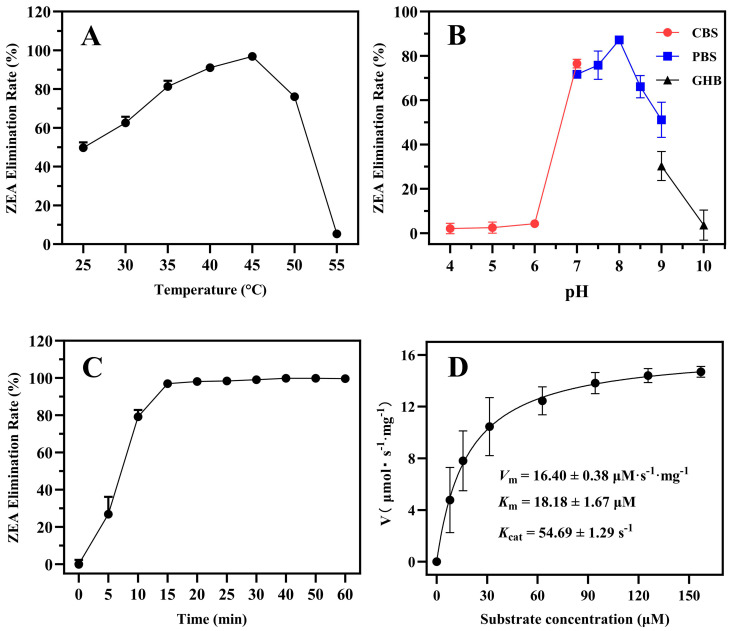
Enzymatic characterization of recombinant protein ZPH1101. Effects of temperature (**A**), pH value (**B**), and reaction time (**C**) on ZPH1101 activity. Kinetic constants for ZPH1101 (**D**). The values of the relative activity are the averages of three replicates, with error bars indicating standard deviations. In panel A, the pH value was 7.0, and the reaction time was 20 min. In panel B, the temperature was 45 °C, and the reaction time was 20 min. In panel C, the pH was 8.0, and the temperature was maintained at 45 °C. In panel D, the temperature and pH were 45 °C and 8.0, respectively. The amount of recombinant ZPH1101 protein used in each assay was 5 μL (1 mg/mL). CBS: citrate buffer saline; PBS: phosphate buffer saline; GHB: glycine-sodium hydroxide buffer.

We investigated the effects of temperature, pH, reaction time, and metal ions on enzyme activity of ZPH1101. The results showed that the ZPH1101 activity initially increased and then decreased across a reaction temperature range of 25 to 55 °C, with an optimal temperature of 45 °C (Figure 3A). ZPH1101 exhibited maximum activity at pH 8.0, with significant decreases in catalytic activity observed below pH 7.0 or above pH 9.0 (Figure 3B). ZPH1101 achieved a conversion rate of over 95% of ZEA within 15 min under optimal temperature and pH conditions (Figure 3C). The enzyme kinetic parameters *V*_max_, *K*_m_, and *k*_cat_ were determined using the Michaelis–Menten model under optimal conditions (Figure 3D), and the calculated values were 16.40, 18.18, and 54.69, respectively.

**Figure 4:**
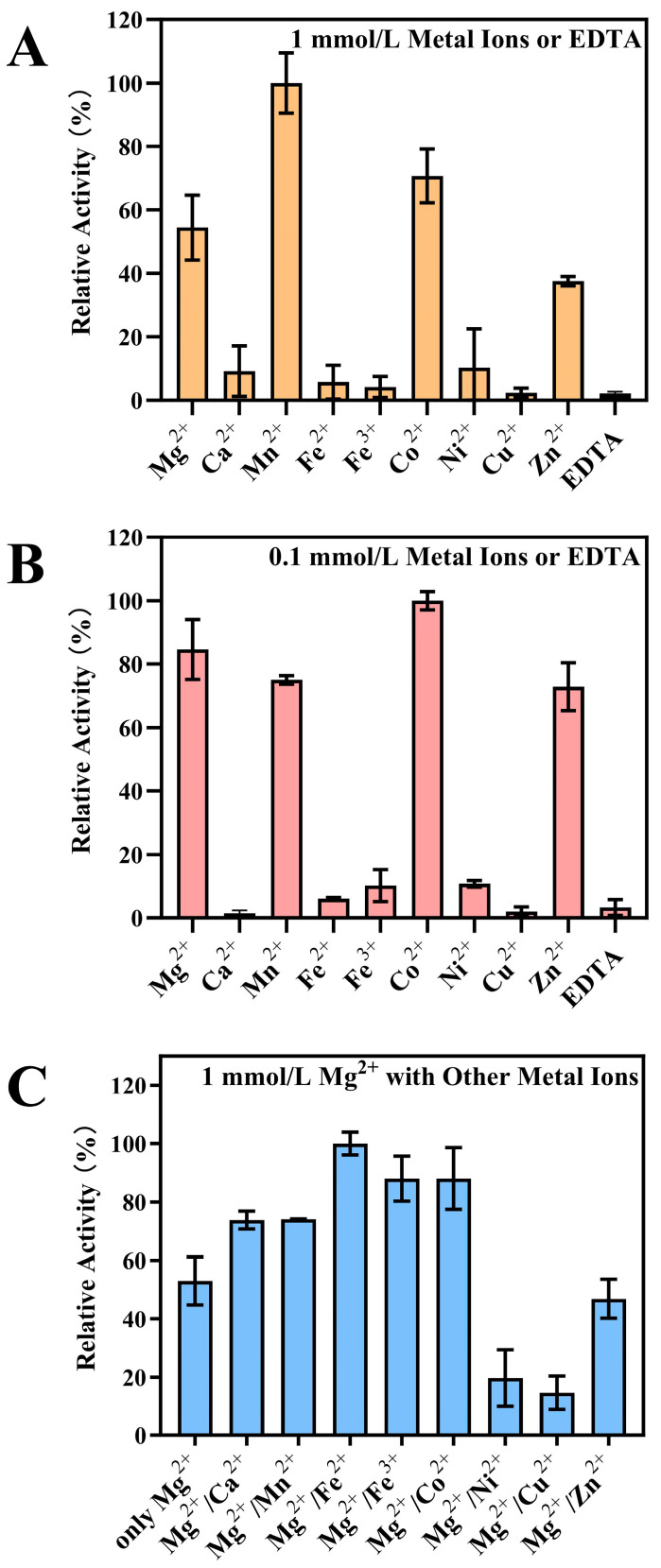
Effects of different concentration of metal ions or EDTA (**A**,**B**), and the coexistence of two metal ions (**C**) on ZPH1101 activity.

To assess the impact of metal ions on ZEA conversion efficiency, among the tested metal ions, Mg^2+^, Mn^2+^, Co^2+^, and Zn^2+^ activated ZPH1101 activity, while other metal ions had no significant effect (Figure 4A,B). Furthermore, we added different types and concentrations of metal cations to a reaction system already containing Mg^2+^, while maintaining all other conditions the same. The result showed that the addition of either Fe^2+^ or Fe^3+^ significantly enhanced the conversion efficiency of ZEA. To reduce potential concentration-related effects, we further investigated this phenomenon by varying the ion concentrations (Figure 4C and Table S2). Our findings showed that at low concentrations (less than 1 mmol/L), there was no clear correlation between the concentrations of the two metal ions and the observed effect.

### 2.5. Sequence and Structure Analysis of ZPH1101

**Figure 5:**
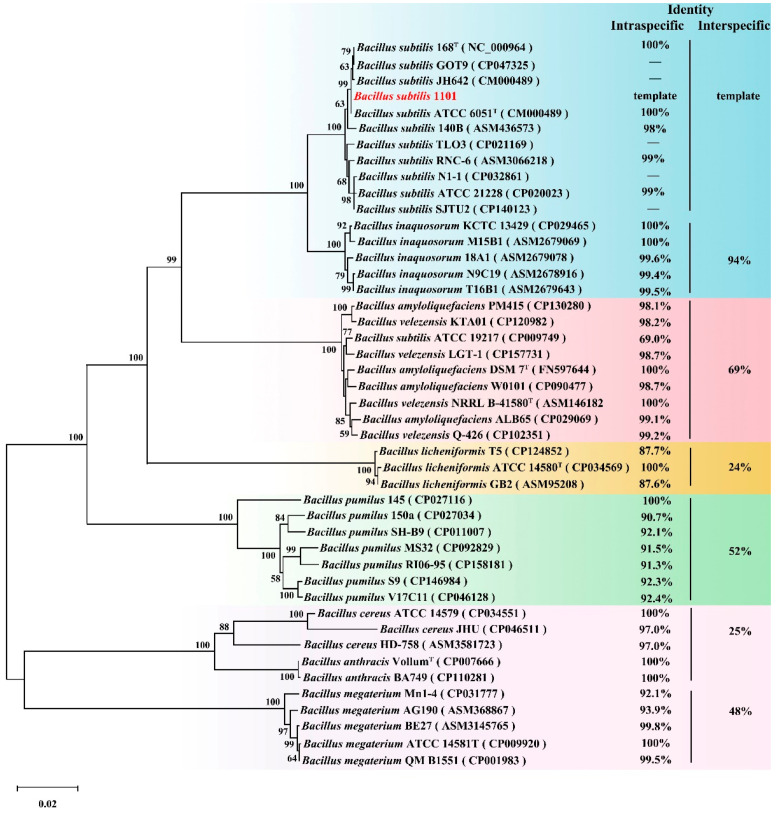
Distribution of ZEA phosphotransferase genes in *Bacillus*. Phylogenetic trees of various *Bacillus* species were constructed based on 16S rRNA and *gyrB*. BLAST was used to perform a homology analysis of ZPH1101 homologous sequences. The first column of data on the right represents the identity of ZEA phosphotransferase within species. The second column of data represents interspecific identity. The red font denotes the source of ZPH1101 in this study.

A phylogenetic tree was constructed using 16S rRNA sequences from a variety of randomly selected *Bacillus* species from the NCBI. The ZEA phosphotransferase gene was found to be widely present across *Bacillus* species and highly conserved within the same species (Figure 5). This suggests that *Bacillus* has an abundance of ZEA phosphotransferase.

**Figure 6:**
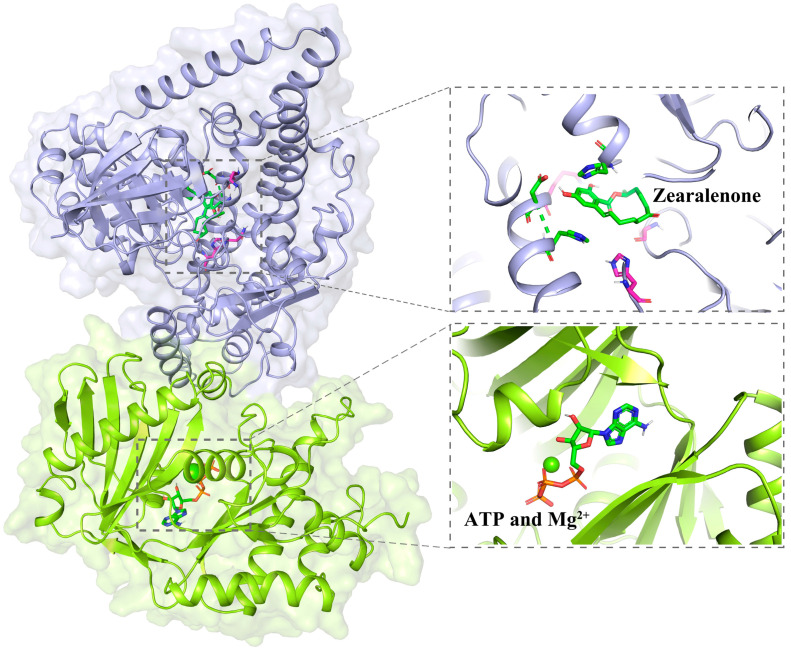
Molecular docking. Structure of the wild-type ZPH1101 in complex with ZEA, ATP, and Mg^2+^. ZEA and ATP are shown as sticks. ZEA binding sites are colored by atom type in magenta and blue. Mg^2+^ is shown as a fluorescent green spherical.

Through a comparison of the amino acid sequences of ZPH1101 and ZPH, we found that they had similar secondary structures (Figure S4). When we docked ZPH1101 with the ZEA molecule (Figure 6), we found that the ZEA could form hydrogen bonds with Asp627, His630, and His795, implying that these three amino acid residues might be responsible for the catalysis of ZEA. To verify this possibility, we mutated each of the three residues to alanine and determined their activities. The results suggest that the D627A, H630A, and H795A mutants lost the ability to phosphorylate ZEA in vitro (Figure S5). In addition, we found that the His795 residue was highly conserved in ZPH1101 and its orthologs, and phosphoenolypyruvate synthase of *Escherichia coli* has similar features [32]. These results suggest that conserved His residue might act as an acceptor and a donor of the phosphate group during ZEA phosphorylation.

### 2.6. Characteristics of Knockout Mutant

**Figure 7:**
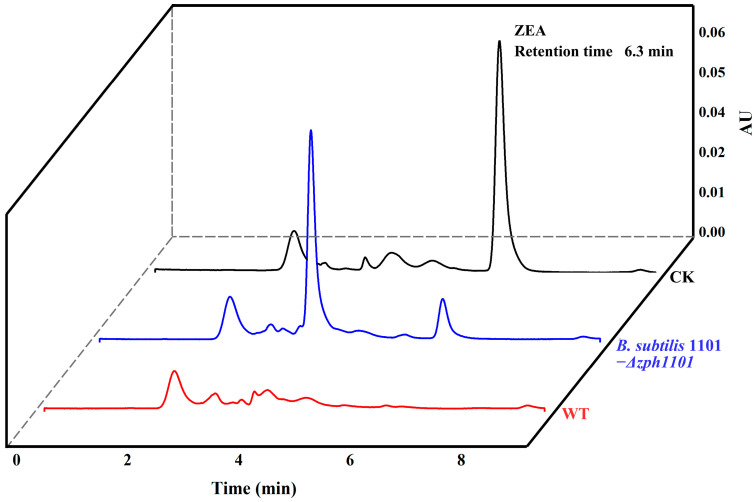
HPLC analysis of residual ZEA after 8 h of biotransformation by wild-type *B. subtilis* 1101 and its knockout mutant. The black line corresponds to the blank control, while the red and blue lines represent the wild-type strain and the knockout mutant, respectively.

The *zph1101* knockout strain 1101-Δ*zph1101* was constructed using suicide plasmid pK18mobsacB. The gene *zph1101* deletion was confirmed by two separate diagnostic PCR amplifications (Figure S6A,B). After 8 h of incubation, the knockout mutant showed an 80% elimination of ZEA, compared to a 99% elimination rate in the wild-type strain (Figure 7). This result indicates that the phosphotransferase pathway accounts for only a small portion of ZEA biotransformation in *B. subtilis* 1101, suggesting the presence of other biotransformation pathways in this strain.

### 2.7. Estrogenic Activity of ZEA-P

**Figure 8:**
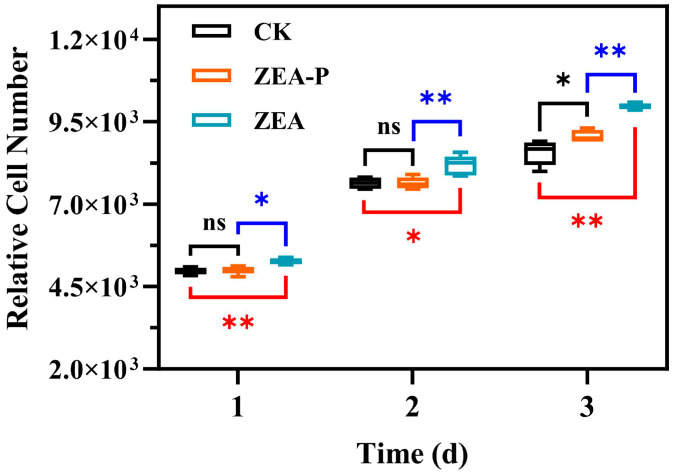
Effects of ZEA and ZEA-P on the proliferation of MCF-7 cells. CK represents the untreated group, while the ZEA and ZEA-P treatment groups were treated at a concentration of 10 nmol/L. Significant differences between treatment groups are indicated as follows: * *p* < 0.05, ** *p* < 0.01; ns, not significant. Each reported value represents the means ± SD (n = 3).

We investigated the effects of 10 nmol/L ZEA and ZEA-P on MCF-7 cell proliferation. The results showed that proliferation in the ZEA-treated group was significantly higher than in the untreated group, but no significant difference was observed between the ZEA-P-treated group and the untreated group (Figure 8).

## 3. Discussion

This study identified *B. subtilis* 1101, which has the potential to rapidly eliminate ZEA. To further elucidate the biotransformation mechanism of ZEA in strain 1101, a phosphotransferase (ZPH1101) was identified from its genome, highlighting its enzymatic characteristics. This work is the first report on the enzymatic characteristics of ZEA phosphotransferase.

Previous studies demonstrated that low-dose ZEA exhibits potent estrogenic activity, leading to reproductive toxicity in both humans and animals [33,34]. In farmed animals, the primary target organs of ZEA are the liver and uterus (or testicles), and its natural metabolic pathway in the body predominantly involves glucuronidation [35]. This modification significantly enhances the water solubility of the substance, facilitating its rapid excretion via urine. In pigs, which are particularly sensitive to ZEA, glucuronidated ZEA can be reduced in the liver and subsequently converted into alpha-zearalenol, a compound that is more estrogenic than ZEA. The resulting alpha-zearalenol is not easily eliminated from the body, circulating repeatedly in the bloodstream and accumulating in estrogen-sensitive reproductive organs [36]. As a result, even low concentrations of ZEA in feed can exacerbate reproductive toxicity in pigs, making them more vulnerable.

In vitro, we utilized estrogen-sensitive MCF-cells to evaluate the estrogenic activity, demonstrating that the estrogenic activity of the biotransformation product (ZEA-P) was significantly lower than that of ZEA. The toxicity of ZEA phosphorylation products exhibited contradictory findings in prior studies [29,37]. Yang et al. utilized estrogen-responsive engineered yeast cells and found that ZEA-P exhibited significantly reduced estrogen toxicity in comparison to ZEA, supporting our findings [29]. However, Asaduzzaman et al. evaluated the estrogenic toxicity of ZEA-P using the human endometrial Ishikawa cell line, concluding that the estrogenic toxicity of ZEA-P remained unmitigated [37]. Notably, ZEA-P was found to be extremely stable under ZPH enzymatic activity, with no detectable conversion back to ZEA or production of additional ZEA derivatives [29]. Furthermore, the water solubility of ZEA-P is much higher than that of ZEA, suggesting that it may be less likely to accumulate in the body and presumably be rapidly excreted in the urine, similar to ZEA glucuronidation products [35,38]. Taken together, our findings reveal that structurally stable ZEA-P has significantly lower toxicity than ZEA, indicating that the phosphorylation pathway is an effective mechanism for the elimination and detoxification of ZEA.

We identified conserved orthologs of ZPH1101 in various *Bacillus* species, and these sequences within the same *Bacillus* species exhibited a high degree of conservation. This indicates that ZEA phosphorylation is a naturally occurring detoxifying process. Thus, there are abundant reserves of phosphotransferases that facilitate the catalysis of ZEA in the natural environment, which provides a possibility for studying the origin and molecular mechanism of the ZEA phosphotransferase gene.

Previous studies have found a variety of enzymes that modify ZEA in diverse organisms, primarily including phosphotransferases, glycoside transferases, and sulfotransferases [24,26,27,28,29]. The catalytic activity of phosphotransferase is dependent on the presence of ATP and Mg^2+^ ions [29,39,40]. Upon comparing with previously published phosphotransferases, we found that the catalytic mechanism of this particular type of enzyme is fundamentally identical. They contain three domains with different functions: ATP and metal ion-binding domain (AD), His domain (HD), and substrate-binding domain (SD) [39,40]. The HD can switch between the AD and SD, binding to each separately, resulting in the formation of two unique protein conformations. The HD-AD conformation promotes ATP binding and hydrolysis, while a His residue in the SD captures the phosphate. The HD-SD conformation initiates the phosphorylation reaction of the substrate. Notably, in metal ion-dependent tests, we found that the combined presence of low concentrations of Mg^2+^ and Fe^2+^ (or Fe^3+^) significantly enhanced the catalytic activity of ZPH1101 towards ZEA; the catalytic efficiency was enhanced by 1.7–1.9-fold relative to Mg^2+^ alone. Previous studies have demonstrated that metal ion-dependent enzymes can be activated synergistically by multiple metal ions [41,42,43]. Despite the fact that comparable effects have been documented in other enzymes, no such effect has been identified in ZEA phosphotransferases. Jing et al. identified calcium-binding proteins that are highly selective for Ca^2+^ and Mg^2+^ [44]. Fukui et al. discovered that the presence of extra divalent metals, including manganese, significantly increased the endonuclease activity of MutL in the zinc-dependent MutL endonuclease reactions [45]. The cooperative effect may be due to the ability of Fe^3+^ or Fe^2+^ to more effectively stabilize the charge following the release of the phosphate group from ATP bound to Mg^2+^ during ATP hydrolysis, thus promoting the transfer of the phosphate group.

We conducted a gene knockout experiment of *zph1101* to investigate the biotransformation pathway of ZEA in strain 1101. The results revealed the presence of extra enzymes or metabolic pathways in *B. subtilis* 1101 that have the ability to modify ZEA. Therefore, further investigation and validation of these biological pathways are essential. Moreover, *B. subtilis* 1101, with its well-defined genetic background and probiotic characteristics, serves as an ideal biomaterial for the biotransformation of ZEA. The utilization of this strain in the development of ZEA-degrading bacterial formulations has substantial potential for practical applications in production.

## 4. Conclusions

This study characterized the ZEA phosphotransferase ZPH1101 from strain *B. subtilis* 1101 and identified its biotransformation product, ZEA-P, which exhibited significantly lower estrogenic toxicity than ZEA. Furthermore, gene knockout experiments further confirmed the presence of additional ZEA biotransformation pathways in *B. subtilis* 1101. This study expands the ZEA biological detoxification enzyme resources and offers further opportunities for researchers working on ZEA enzymatic detoxification work.

## 5. Materials and Methods

### 5.1. Samples, Chemicals, Strains, and Plasmid

The samples used for isolation and screening of ZEA-degrading bacteria were collected from livestock and poultry manure in Liuhe District, Jiangsu Province, China. ZEA (purity > 98%) was purchased from Romer Labs (Beijing, China). A standard stock solution of ZEA was prepared by diluting it in acetonitrile. The ClonExpress II one-step cloning kit was acquired from Vazyme Biotech Co., Ltd. (Nanjing, China). The formulations of minimal salt medium (MSM) and Luria–Bertani (LB) medium were described by Hu et al. [13]. Isopropyle β-D-1-thiogalactopyranoside (IPTG) was purchased from Yuanye Bio-Technology Co., Ltd. (Shanghai, China). The strains and plasmids utilized in this study are listed in Table S3.

### 5.2. Isolation and Identification of Strain 1101

A 1 g portion of the manure sample was measured and mixed with 10 mL of MSM medium containing 10 μg/mL ZEA. The resulting mixture was incubated at 30 °C and 180 rpm for five days. For sample pretreatment, 0.5 mL of the culture medium was collected and combined with an equal volume of ethyl acetate to extract the ZEA. The extract was dried under a nitrogen stream to remove the ethyl acetate before an equal volume of chromatographic methanol was added. Then, the concentration of ZEA in the bacterial culture was determined using high-performance liquid chromatography (HPLC).

The mixed bacterial solutions with a decreased ZEA concentration were diluted, spread on LB plates, and incubated upside down at 30 °C. Individual colonies were chosen for subsequent examination, and this procedure was repeated until a pure bacterium was obtained. By employing the aforementioned procedure for repeated confirmation, a strain denoted as 1101, capable of rapidly degrading ZEA, was isolated. Strain 1101 was cultivated by streaking on LB plates, and its colony phenotype was examined visually. Multiple sequence alignment was conducted using MEGA X (version 10.2.5), and a neighbor-joining phylogenetic tree was constructed by concatenating the 16S rRNA and *gyrB* gene sequences. Evolutionary distances were estimated using Kimura’s two-parameter model [46].

To eliminate the possibility of ZEA biosorption, we conducted a control experiment. One mL of fermentation broth (A_600_ = 1.0) and the sterile fermentation supernatant were combined with 9 mL of MM medium (pH 7.0) containing 10 μg/mL ZEA. The same volume of sterile water was used as a blank control, and an additional control treatment was implemented: The harvested cells were inactivated via high-temperature treatment before incubation. Following incubation at 30 °C for 1, 2, 4, and 8 h, 0.5 mL of the reaction mixture was collected from each treatment for pretreatment. The residual ZEA concentration in all treatments was then quantified using HPLC.

### 5.3. Growth and Biotransformation Characteristics of Strain 1101

#### 5.3.1. Determination of Optimal Growth Temperature and pH

A single colony from a fresh plate of strain 1101 was inoculated into 20 mL of LB medium with pH 6.8. The culture was incubated at 30 °C and 180 rpm until the optical density of the bacteria at 600 nm reached a range of 0.6–0.8, thereby yielding the seed solution. The seed solution was inoculated into 100 mL of LB medium with a 1% inoculum. The cultures were incubated for 36 h at 25 °C, 30 °C, and 37 °C, with constant pH and rotation speed. The cultures were incubated at pH levels of 5.0, 6.0, 7.0, 8.0, and 9.0 while maintaining the same temperature and rotation speed. Samples were taken at intervals of 4 h, and the A_600_ values were measured using a UV-VIS spectrophotometer (UV-19001, Shimadzu, Japan).

#### 5.3.2. Determination of Optimal Biotransformation Temperature and pH

The seed solution was inoculated into 100 mL of LB medium containing 10 μg/mL ZEA, with a 5% inoculum. In order to determine the optimal reaction temperature for biotransformation, the cultures were subjected to incubation for 36 h at three different temperatures: 25 °C, 30 °C, and 37 °C. Similarly, to explore the optimal reaction pH for biotransformation, the cultures were incubated at pH levels of 5.0, 5.5, 6.0, 7.0, and 8.0. Simultaneously, LB with the same amount of ZEA but lacking bacteria served as the control. Each experimental group was set with three replicates, and samples were taken every 12 h. Following sample pretreatment, the ZEA concentration was determined by HPLC.

### 5.4. Genome Sequencing and Annotation

The draft genome sequence of strain 1101 was generated by Shanghai Biozeron Biotechnology Co., Ltd. (Shanghai, China) using the Illumina NovaSeq 6000 sequencing platform. The unprocessed paired-end reads were subjected to trimming and quality control procedures using Trimmomatic (version 0.36). The resulting cleaned data were subsequently utilized for further analysis. The ABySS assembler (version 2.2.0) was employed with GapCloser (version 1.12) to generate the final assembly. The gene models for strain 1101 were predicted using the ab initio method and then identified with GeneMark. The blastp function was then used to annotate all of the predicted gene models in the Nr, Swiss-Prot, KEGG, and COG databases.

### 5.5. Gene Cloning and Expression

Using a local blast, we found a homologous sequence of the ZPH gene of *B. subtilis* Y816 [29] in the genome of strain 1101. We hypothesized that it functions similarly to ZPH based on gene annotation data and have designated it ZPH1101. The 1101 genomic DNA was extracted using the Tianamp Bacteria DNA Kit (Tiangen Biochemical Technology Co., Ltd., Beijing, China), and the primer pairs used to amplify *zph1101* from the genome are listed in Table S1. The *zph1101* gene was cloned into the pET29a vector, and the resulting recombinant plasmid was transformed into *E. coli* BL21(DE3) using the heat shock method to induce intracellular expression [47,48,49].

The constructed *E. coli* BL21(DE3) strain was used for inducible expression of recombinant ZPH1101, with the resultant enzyme containing an N-terminal 6 × His tag. After reaching the A_600_ of 0.6–0.8, 0.5 mmol/L IPTG was added to the system, and then the culture was maintained at 20 °C and 180 rpm for 12 h. The harvested cell pellet was then resuspended in PBS (pH 7.0) buffer and disrupted by sonication at 300 W for 15 min. Subsequently, the crude enzyme solution was obtained by centrifugation at 10,000× *g* for 10 min at 4 °C. It was then filtered through a 0.45 μm membrane to remove bacterial debris. The recombinant ZPH1101 was purified using the NGC™ Scout 10 Plus system (Bio-Rad, Hercules, CA, USA) that was equipped with a HisTrap HP Ni-NTA column (Cytiva, Marlborough, MA, USA). During the loading phase, recombinant ZPH1101 was immobilized on the Ni-NTA column at a flow rate of 1 mL/min. Afterwards, 4 column volumes of elution buffer containing 80 mmol/L imidazole were used for elution to remove impurity protein. Then, 4 column volumes of elution buffer containing 250 mmol/L imidazole were used to elute and collect ZPH1101. The concentration of purified protein was determined using the BCA assay kit (Tiangen Biotech Co., Ltd., Beijing, China).

### 5.6. Knockout of the Gene zph1101

Gene knockout was performed using homologous recombination. The recombinant suicide plasmid pK18mobsacB containing the upstream and downstream regions of the *zph1101* was constructed and transformed into the competent cell of *B. subtilis* 1101 using the Spizizen transformation method [50]. The transformed cells were subsequently distributed on an LB plate containing kanamycin. The single-exchange strain was obtained after selecting positive clones and verifying them using colony PCR. The accurate single-exchange strain was cultivated, diluted, and spread on an LB plate supplemented with 20% sucrose. Following the second colony PCR, the double-exchange strain was identified as a positive clone, indicating that the knockout process was successful.

In order to evaluate whether the phosphotransferase pathway is the exclusive pathway for the elimination of ZEA by *B. subtilis* 1101, the elimination rates of ZEA were compared between the knockout mutant and wild-type strains over the same time period. The cultures of both strains were inoculated into MSM containing 10 μg/mL of ZEA at a 10% addition rate, independently. ZEA concentrations remaining after incubation at 30 °C for 8 h were determined using HPLC.

### 5.7. Enzyme Activity Assay

The ZPH1101 activity was quantified as one unit (U), which is the amount of enzyme required to convert 1 μmol of ZEA per min under optimal conditions. Each reaction system contained 10 μg/mL ZEA, 5 μL of ZPH (1 mg/mL), 1 mmol/L ATP, and 1 mmol/L Mg^2+^ in a total volume of 0.25 mL. Following mixing the reaction systems, they were incubated at 37 °C for 10 min, and then an equal volume of ethyl acetate was added to terminate the reaction. The residual ZEA in the reaction system was subsequently extracted and detected using HPLC. We investigated the enzymatic properties of recombinant ZPH1101 at temperatures ranging from 25 to 55 °C and pH ranges of 4.0 to 10.0. The effects of potential activators and inhibitors on ZPH1101 activity were evaluated by adding various types of metal ions (Mg^2+^, Ca^2+^, Mn^2+^, Fe^2+^, Fe^3+^, Co^2+^, Ni^2+^, Cu^2+^, Zn^2+^) and EDTA.

### 5.8. Sequence Analysis and Molecular Docking

ClustalW (version 2.1) was used for protein sequence alignments in the phylogenetic analysis of ZPH1101 [51]. Espript 3.0 was utilized to improve the graphical sequence alignment [52]. The structural model of ZPH1101 was constructed by SWISS-MODEL [53], and the 3D models of ZEA were obtained from PubChem (https://pubchem.ncbi.nlm.nih.gov, accessed 26 June 2024). Molecular docking was performed using AutoDock software (version 4.2.6) [54], and PyMOL software (version 2.5.5) was used for image processing.

### 5.9. Detection of ZEA and Its Biotransformation Products

The ZEA content was determined using HPLC (Waters e2695, Waters Corp., Milford, MA, USA) with a UV detector at a wavelength of 236 nm. Chromatographic separation was performed on an Eclipse XDB-C18 column (ODS, 4.6 mm × 250 mm, Zorbax) using water–methanol (20:80, *v*/*v*) as the eluent at a flow rate of 0.8 mL/min. The quantification of ZEA was performed using the external standard method, and all experimental data were presented as the mean ± standard deviation (SD) from three replicates. The ZEA biotransformation rate was calculated according to the following formula:

*B* = (1 − *Ct*/*Co*) × 100%

where *B* is the biotransformation rate of ZEA, and *Co* and *Ct* are the ZEA concentrations in the control group and experimental treatments, respectively.

To identify ZEA biotransformation products, strain 1101 was inoculated into 10 mL of MSM containing 10 μg/mL ZEA, and the fermentation broth was collected after 24 h and lyophilized. For mass spectrometry analysis, the products were dissolved in 2 mL of methanol and then filtered through a 0.22 mm filter. The LC-TOF-MS/MS analysis was performed using an AB SCIEX Triple-TOFTM 5600 system (Ontario, ON, Canada). TOF-MS/MS scans were performed in the mass range of 100–1000 Da with a collision energy (CE) of 40 eV.

### 5.10. Determination of the Estrogenic Toxicity of ZEA and Its Biotransformation Products

To evaluate the estrogenic toxicity of ZEA and its biotransformation products, we assessed MCF-7 cell proliferation using the CCK-8 assay. MCF-7 cells were inoculated in 96-well plates at 5 × 10^3^ cells/well in 100 μL of DMEM supplemented with 10% estrogen-free FBS, 100 IU/mL penicillin, and 100 μg/mL streptomycin. After allowing the cells to adhere for 24 h, the medium was replaced with DMEM containing 10 nmol/L ZEA and its biotransformation products. The absorbance values were measured at 490 nm using a microplate reader after 24, 48, and 72 h of incubation, respectively. Each experiment was performed in triplicate.

## Data Availability

The data presented in this study are available on request from the corresponding author due to (the authors need to determine whether the request is reasonable).

## Supplementary Materials

The following supporting information can be downloaded at: https://www.mdpi.com/article/10.3390/toxins17010021/s1, Figure S1. Phenotype of strain 1101 (A) and agarose gel electrophoresis of 16S rRNA and *gyrB* (B). Colony morphology of strain 1101 cultured on LB plate at 30°C for 24 h. The lengths of amplified 16S rRNA and *gyrB* were 1550 bp and 1250 bp, respectively. Phylogenetic tree (C) of strain 1101 based on 16S rRNA and *gyrB* sequences, illustrating the relationship of strain 1101 to related species. Nodes display percent bootstrap values (1000 replicates) that are larger than 50%. Elimination ability of strain 1101 to ZEA under different treatment conditions (D). CK, FB, FS and HT represent blank control, fermentation broth, fermentation supernatant and high temperature inactivation treatment, respectively; Figure S2. The growth characteristics of strain 1101 in different conditions. Growth curves of strain 1101 at various temperatures (A) and pH levels (B), where LB medium was used to cultivate strain 1101. Error bars represent standard deviations; Figure S3. SDS-PAGE analysis of the purified recombinant ZPH1101. The target recombinant protein (90 kDa) was identified on the SDS-PAGE gel by comparison with molecular weight markers, with its band appearing within the expected range between the 95 kDa and 72 kDa standards; Figure S4. Sequence alignment of ZPH1101 and ZPH. The squiggles denote α-helices and β-strands are rendered as arrows, strict β-turns as TT letters, while the η symbol refers to a 3_10_-helix. The three residues with catalytic ZEA function are marked by blue boxes and located in the 23rd and 29th α-helices respectively; Figure S5. Determination of enzyme activity of ZPH1101 mutants in vitro. The wild-type strain 1101 (WT) is represented in black, while the three mutants, D627A, H630A, and H795A, are shown in blue, green, and red, respectively. Error bars represent standard deviations; Figure S6. PCR analyses for the knockdown of zph1101 in strain 1101 (A & B). A: lane M1 is a DNA marker (TaKaRa Co., Ltd., catalog No. 3427Q); lane N1 is the negative control with water as the PCR template; lane P1 is the positive control with plasmid pK18mobsacB-Δzph1101 as the PCR template; lane S1 is the tested sample with 1101-pK18mobsacB-Δzph1101 genomic DNA as the PCR template, and all of the above lanes were used to amplify the sacB gene using the sacB-F/R primer pair. B: lane M2 is a DNA marker (TaKaRa Co., Ltd., catalog No. 3428Q); lane N2 is the negative control with water as the PCR template; lane P2 is the positive control with genomic DNA of strain 1101 as the PCR template; lane S2 is the tested sample with Δzph1101-1101 genomic DNA as the PCR template, and all of the above lanes were used to amplify the zph1101 gene using the zph1101-F/R primer pair; Table S1. Primers used in this study; Table S2. ZEA transformation rate under different concentrations of two metal ions; Table S3. Strains and plasmids used in this study.
