# Supplementary material for: Characterization of the Phosphotransferase from Bacillus subtilis 1101 That Is Responsible for the Biotransformation of Zearalenone
Source: Toxins (Basel). 2025 Jan 3;17(1):21. doi: 10.3390/toxins17010021 (PMC11768653; doi:10.3390/toxins17010021)
Supplement: Supplementary file 1 [file toxins-17-00021-s001.zip › toxins-3369686-supplementary.pdf]

Supplemental Materials

Characterization of the Phosphotransferase from *Bacillus subtilis* 1101 That Is Responsible for the Biotransformation of Zearalenone

Yuzhuo Wu, Qiuyu Zhou, Junqiang Hu, Yunfan Shan, Jinyue Liu, Gang Wang, Yin-Won Lee, Jianrong Shi and Jianhong Xu

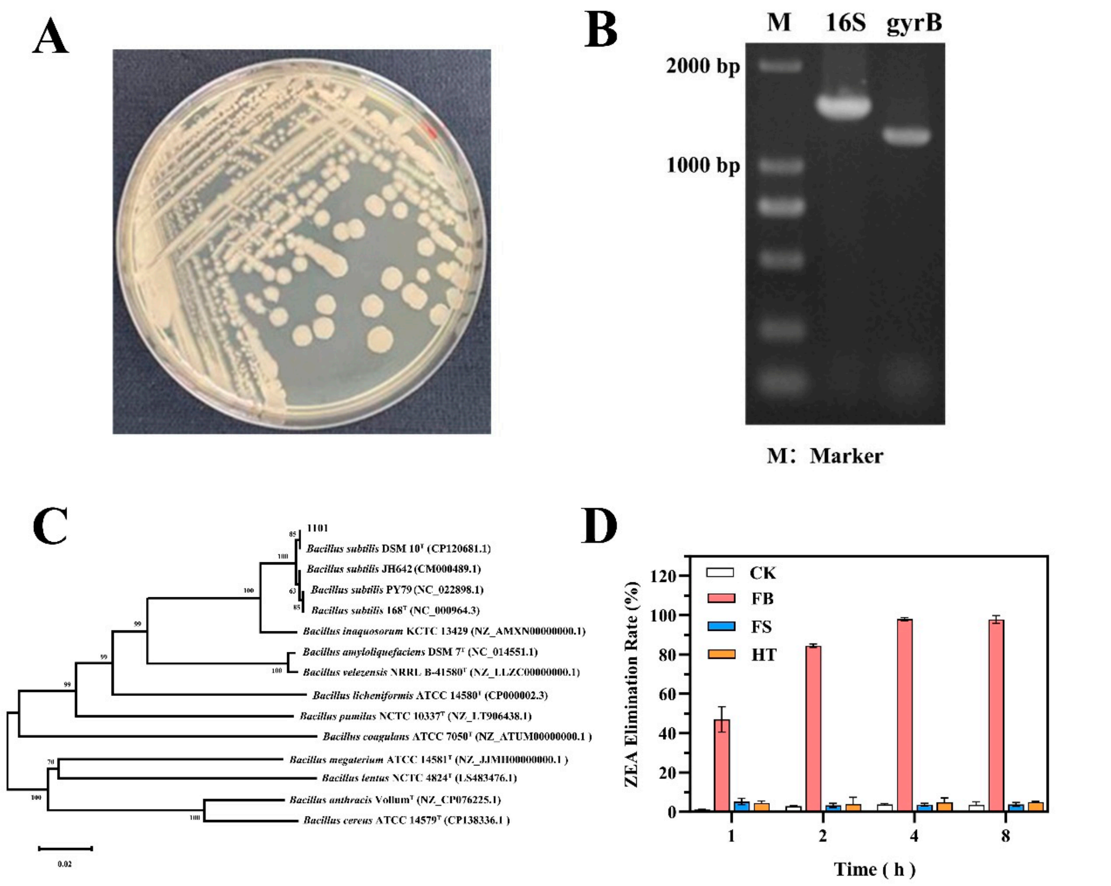

Figure S1. Phenotype of strain 1101 (A) and agarose gel electrophoresis of 16S rRNA and *gyrB* (B). Colony morphology of strain 1101 cultured on LB plate at 30°C for 24 h. The lengths of amplified 16S rRNA and *gyrB* were 1550 bp and 1250 bp, respectively. Phylogenetic tree (C) of strain

1101 based on 16S rRNA and *gyrB* sequences, illustrating the relationship of strain 1101 to related species. Nodes display percent bootstrap values (1000 replicates) that are larger than 50%. Elimination ability of strain 1101 to ZEA under different treatment conditions (D). CK, FB, FS and HT represent blank control, fermentation broth, fermentation supernatant and high temperature inactivation treatment, respectively.

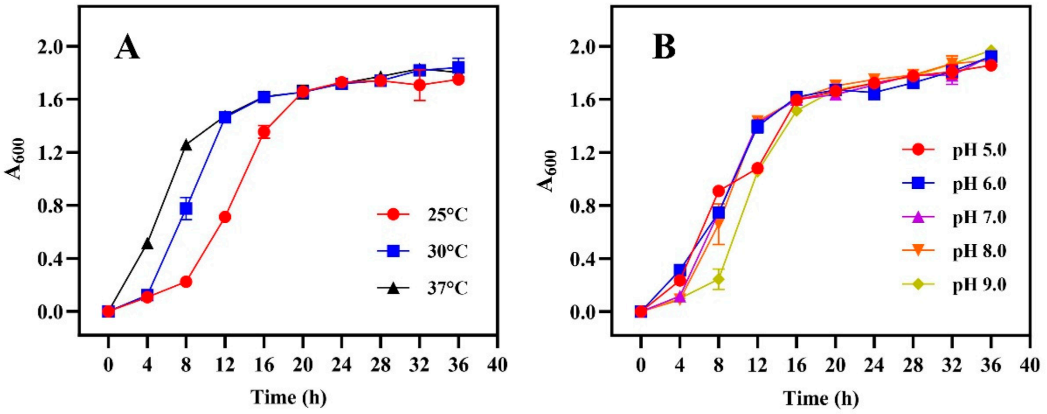

Figure S2. The growth characteristics of strain 1101 in different conditions. Growth curves of strain 1101 at various temperatures (A) and pH levels (B), where LB medium was used to cultivate strain 1101. Error bars represent standard deviations.

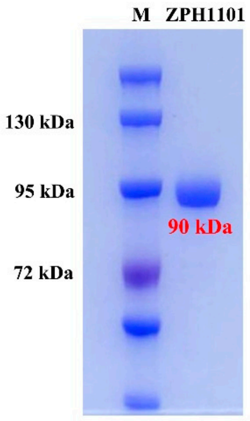

Figure S3. SDS-PAGE analysis of the purified recombinant ZPH1101. The target recombinant protein (90 kDa) was identified on the SDS-PAGE gel by comparison with molecular weight markers, with its band appearing within the expected range between the 95 kDa and 72 kDa standards.

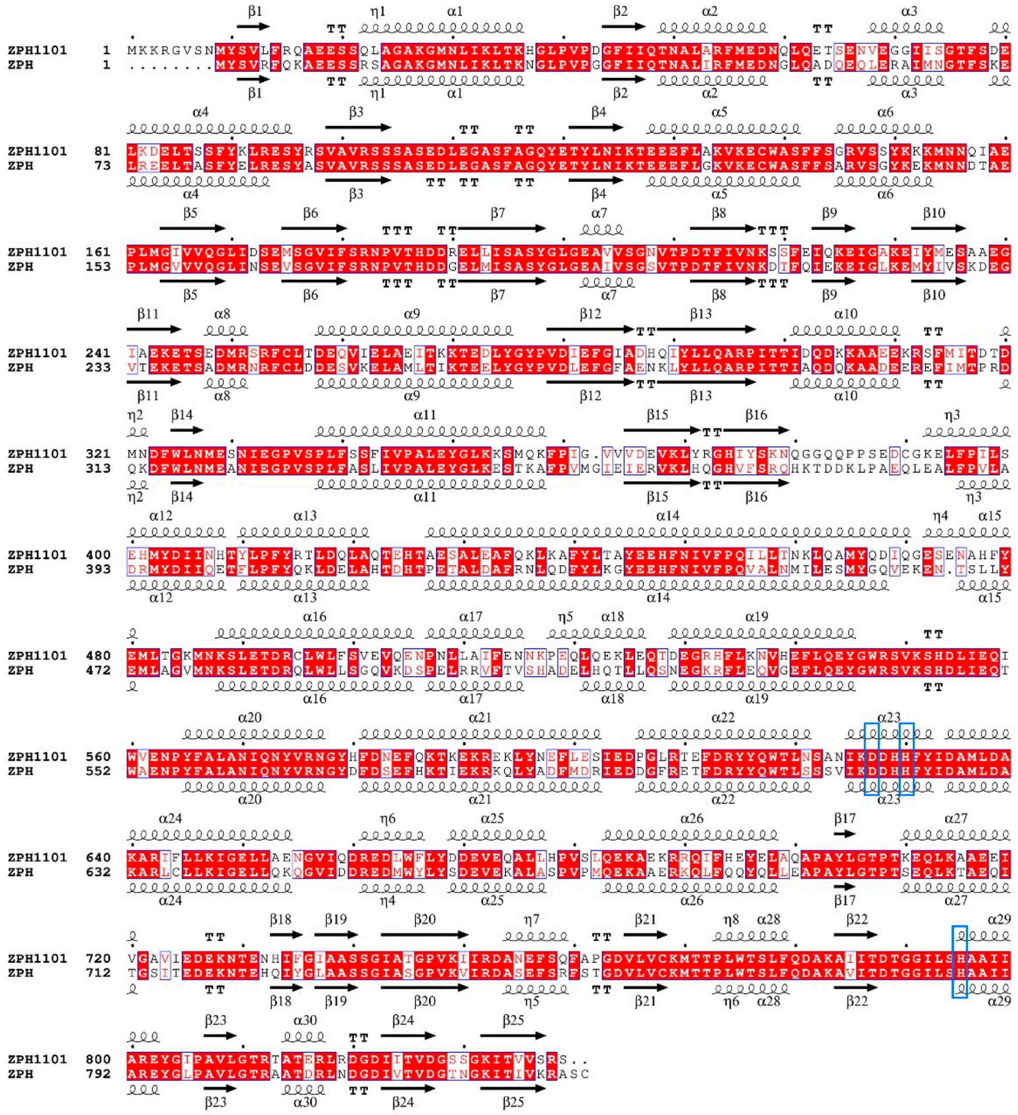

Figure S4. Sequence alignment of ZPH1101 and ZPH. The squiggles denote α-helices and β-strands are rendered as arrows, strict β-turns as TT letters, while the η symbol refers to a 3_{10}-helix. The three residues with catalytic ZEA function are marked by blue boxes and located in the 23rd and 29th α-helices respectively.

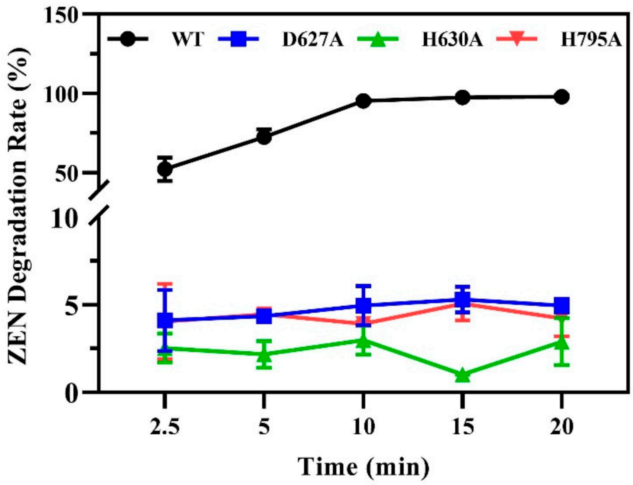

Figure S5. Determination of enzyme activity of ZPH1101 mutants *in vitro*. The wild-type strain 1101 (WT) is represented in black, while the three mutants, D627A, H630A, and H795A, are shown in blue, green, and red, respectively. Error bars represent standard deviations.

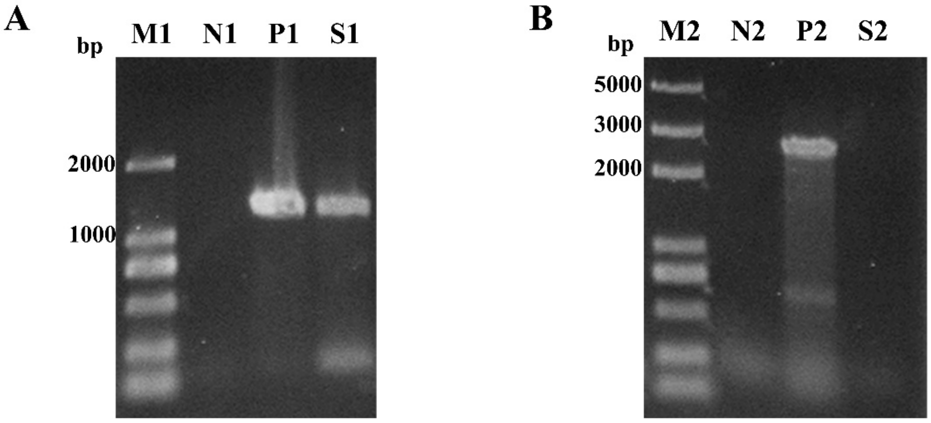

Figure S6. PCR analyses for the knockdown of *zph1101* in strain 1101 (A & B).

A: lane M1 is a DNA marker (TaKaRa Co., Ltd., catalog No. 3427Q); lane N1 is the negative control with water as the PCR template; lane P1 is the positive control with plasmid pK18mobsacB-Δ*zph1101* as the PCR template; lane S1 is the tested sample with 1101-pK18mobsacB-Δ*zph1101* genomic DNA as the PCR template, and all of the above lanes were used to amplify the *sacB* gene using the *sacB*-F/R primer pair.

B: lane M2 is a DNA marker (TaKaRa Co., Ltd., catalog No. 3428Q); lane N2 is the negative control with water as the PCR template; lane P2 is the positive control with genomic DNA of strain 1101 as the PCR template; lane S2 is the tested sample with Δ*zph1101*-1101 genomic DNA as the PCR template, and all of the above lanes were used to amplify the *zph1101* gene using the *zph1101*-F/R primer pair.

Table S1. Primers used in this study

Primer pairs	Sequence (5'–3')	Description
27F	AGAGTTTGATCCTGGCTCAG	To amplify the 16S rRNA gene
1492R	GGTTACCTTGTTACGACTT	
gyrB-F	AGCAGGATACGGATGTGCGAGCCRTCNACRTCNGCRTCNGTCAT	To amplify the *gyrB* gene
gyrB-R	GAAGTCATCATGACCGTTCTGCAYGCNGGNGGNAARTTYGA	
zph1101-F	AACTTTAAGAAGGAGATATAATGAAGAAAAGAGGGGTTTCAAATATG	To amplify the *zph1101* gene with pET29A homology arms
zph1101-R	GTGCTCGAGTGCGGCCGCTCAGGACCGGCTGACAACT	
L-F	CAGCTATGACATGATTACGTTCCTGTTGCACCCAGC	To amplify the upstream region of *zph1101*
L-R	ATTTGAAACCCCTCTTTTCTTCATTC	
R-F	AAAAGAGGGGTTTCAAATTCCTGATGCGTCCCCC	To amplify the downstream region of *zph1101*
R-R	AAAACGACGGCCAGTGCCCTGCAATGCCCGAAAGAC	
sacB-F	CGAACCAAAAGCCATATAAGGAAAC	To amplify the *sacB* gene
sacB-R	CTGTTAATTGTCCTTGTTCAAGGATG	

Mixed base symbols: "N" indicates C and T; "R" indicates A and G; "Y" indicates G and T.

Table S2. ZEA transformation rate under different concentrations of two metal ions

Ion concentration (mmol/L)	10^{-1} Mg^{2+}	10^{-2} Mg^{2+}	10^{-3} Mg^{2+}	10^{-4} Mg^{2+}
10^{-1} Fe^{3+}	88.01 ± 0.57 c	43.43 ± 0.83 a	98.89 ± 0.95 d	98.42 ± 0.89 d
10^{-2} Fe^{3+}	98.99 ± 0.14 d	67.09 ± 1.05 b	98.72 ± 1.06 d	98.54 ± 1.16 d
10^{-3} Fe^{3+}	98.46 ± 0.33 d	60.72 ± 1.29 b	98.76 ± 1.23 d	98.23 ± 1.40 d
10^{-4} Fe^{3+}	99.03 ± 0.08 d	63.69 ± 1.49 b	98.64 ± 1.07 d	95.41 ± 1.32 d
10^{-1} Fe^{2+}	99.68 ± 0.32 d	92.68 ± 1.02 d	99.82 ± 0.18 d	99.61 ± 0.39 d
10^{-2} Fe^{2+}	99.92 ± 0.08 d	98.65 ± 1.35 d	99.56 ± 0.44 d	99.52 ± 0.48 d
10^{-3} Fe^{2+}	98.65 ± 1.35 d	98.73 ± 1.27 d	99.71 ± 0.29 d	99.85 ± 0.15 d
10^{-4} Fe^{2+}	98.41 ± 1.59 d	98.21 ± 1.79 d	99.33 ± 0.67 d	99.56 ± 0.44 d

Data in the table are shown as mean ± standard deviation. Values with different lowercase letters (a, b, c, d) are significantly different at $p<0.05$ according to the t test.

Table S3. Strains and plasmids used in this study

Strain or plasmid	Description	Source
**Strain**		
Bacillus subtilis 1101	Wild type	From livestock and poultry manure
1101-pK18mobsacB-Δ*zph1101*	Strain 1101 integrated the suicide plasmid pK18mobsacB-Δ*zph1101*	**This study**
Δ*zph1101*-1101	Strain 1101 with knockout of the *zph1101* gene	**This study**
Escherichia coli BL21(DE3)	F^- *ompT hsdS* (r_B^- m_B^-) *gal dcm lacY1* (DE3)	Sangon Biotech Co., Ltd.
E. coli DH5α	F^- *recA1 endA1 thi-1 supE44 relA1 deoR Δ(lacZYA-argF)U169 Φ80dlacZ*ΔM15	Sangon Biotech Co., Ltd.
E. coli BL21(DE3)-pET29a-*zph1101*	*E. coli* BL21(DE3) harboring the plasmid pET29a-*zph1101*	**This study**
**Plasmids**		
pK18mobsacB	pK18mobsacB is a SacB suicide plasmid, Km^r	Preserved in lab
pK18mobsacB-Δ*zph1101*	pK18mobsacB derivative carrying the upstream and downstream regions of *zph1101*, Km^r	**This study**
pET29a (+)	Expression vector, Km^r	Preserved in lab
pET29a-*zph1101*	pET29a (+) derivative carrying *zph1101*, Km^r	**This study**

Km^r, kanamycin resistant.
